# Identification, Typing, Antifungal Resistance Profile, and Biofilm Formation of *Candida albicans* Isolates from Lebanese Hospital Patients

**DOI:** 10.1155/2014/931372

**Published:** 2014-06-01

**Authors:** Ibrahim Bitar, Roy A. Khalaf, Houda Harastani, Sima Tokajian

**Affiliations:** Department of Natural Sciences, School of Arts and Sciences, Lebanese American University, P.O. Box 36, Byblos, Lebanon

## Abstract

As leading opportunistic fungal pathogens identification and subtyping of *Candida* species are crucial in recognizing outbreaks of infection, recognizing particularly virulent strains, and detecting the emergence of drug resistant strains. In this study our objective was to compare identification of *Candida albicans* by the hospitals through the use of conventional versus identification based on the ITS (Internal Transcribed Spacer) and to assess biofilm forming capabilities, drug resistance patterns and correlate these with MLST typing. ITS typing revealed a 21.2% hospital misidentification rate. Multidrug resistance to three drugs out of four tested was detected within 25% of the isolates raising concerns about the followed treatment regimens. Drug resistant strains as well as biofilm formers were phylogenetically related, with some isolates with significant biofilm forming capabilities being correlated to those that were multidrug resistant. Such isolates were grouped closely together in a neighbor-joining tree generated by MLST typing indicating phylogenetic relatedness, microevolution, or recurrent infection. In conclusion, this pilot study gives much needed insight concerning *C. albicans* isolates circulating in Lebanese hospitals and is the first study of its kind correlating biofilm formation, antifungal resistance, and evolutionary relatedness.

## 1. Introduction

As the leading opportunistic fungal pathogen* Candida* infections have increased significantly worldwide, with the species* C. albicans* responsible for most of these infections [1–3]. Over the past two decades,* Candida* species have become the leading pathogens responsible for nosocomial bloodstream infections with* C. albicans* causing more than 50% of these infections [4].* C. albicans*, a dimorphic commensal yeast, has two reservoirs: the patients' normal flora and the environment. Both interact making it difficult to block transmission of the pathogen between patients [5]. Infections range from superficial, affecting the skin, mouth, and vagina, to systemic associated with high morbidity and mortality rates in immunosuppressed individuals, HIV patients, chemotherapy patients, and organ transplant patients [6].

Virulence of* C. albicans* can be attributed to several factors such as phenotypic switching, dimorphic transition between hyphae and yeast, adhesins, and secretion of proteases and phospholipases [7, 8]. The ability of* C. albicans* to dimorph between two main shapes, a round budding yeast and an elongated parallel-walled true hypha (with an intermediary, pseudohyphal form consisting of stretched ellipsoid cells), is the basis of the germ tube test utilized in most hospitals to identify* C. albicans* from other* Candida* species (*C. dubliniensis* being the exception as it can form true hyphae) [6]. Biofilm formation is another important aspect of* C. albicans* pathogenesis. This phenomenon allows* Candida* to adhere to mucosal cells and to plastic surfaces of medical devices such as catheters and dentures leading to device associated infections and eventually spreading nosocomial infections. Biofilm forming cells are phenotypically different from floating cells in that they are embedded in a three-dimensional structure and can proliferate in healthy individuals surviving within the immune system of the host and having an increased resistance to antifungal drugs [8, 9].

Treatment of* Candida* infections in general and* C. albicans* in particular is limited to the availability of classes and number of antifungal drugs. Only four major classes of antifungal drugs are currently available including the most commonly used azoles, polyenes, fluoropyrimidines, and the newly generated echinocandins. The latter is used as an alternative for isolates showing resistance to the former antifungal drugs [10, 11].

As infections caused by* C. albicans* increased worldwide, identification became a must. Conventional methods are unable to identify yeast species within an acceptable error range [12]. As such molecular typing methods were developed including restriction fragment length polymorphisms (RFLP), pulsed-field gel electrophoresis (PFGE), multilocus sequence typing (MLST), and Internal Transcribed Spacer (ITS) sequencing [3, 10].

Overall, antifungal drug resistance (ADR) and fungal virulence characteristics such as biofilm formation are critical issues for the host-pathogen relationship in candidiasis. However, very little is known about the relationship between drug resistance and virulence of* C. albicans* [13]. In this study 85 isolates were collected from two major hospitals in Beirut/Lebanon between June and October 2011. Isolates were identified using API, germ tube, CHROMagar, and ITS sequencing. Furthermore, antifungal susceptibility testing against four antifungal drugs was performed, and the isolates were additionally tested for biofilm formation followed by MLST typing for selected isolates to determine the epidemiological relatedness of the isolates.

## 2. Materials and Methods

### 2.1. Clinical Isolates

A total of 85 clinical* Candida* isolates collected between June 2010 and October 2011 (16 months) were kindly provided by two major hospitals in Beirut. For the sake of confidentiality the hospitals will be referred to as hospital “A” and hospital “B.” Thirty-one samples (36.4%) were recovered from urine, 23 samples (27.1%) from sputum, 12 samples (14.2%) from tracheal aspirates, 11 samples (12.9%) from bronchial lavages, three samples (3.5%) from body fluids, two samples (2.3%) from abscesses, two samples (2.4%) from puss swabs, and one sample (1.2%) from an abdominal swab. 56.4% (*n* = 48/85) of the patients were females while 43.6% (*n* = 37/85) were males (range from two years to 92 years old). Samples were classified by both hospitals as either* C. albicans* or* C.* non-*albicans*.

Germ tube, a test that shows the ability of the isolates to germinate inside a tube, was performed for all the 85 samples. Following three hours of incubation in serum at 37°C, samples were examined under the microscope for their ability to germinate. An isolate is designated as* C. albicans* only if it appeared filamentous upon visualization. The samples were streaked on Potato Dextrose Agar (PDA) and stored in cryobank vials at −80°C until use.

### 2.2. Samples Identification Using Color Forming* Candida* CHROMagar

Clinical isolates were cultured on color forming* Candida* CHROMagar, the CandiSelect 4 (Bio-Rad, Hercules, CA, USA), and incubated at 28°C for 24–48 h according to the manufacturer's instructions.* Candida* species are then identified according to the color of the colony.

### 2.3. Samples Identification Using API 20 C AUX

Fresh colonies were collected after culturing on PDA for 48 h at 28°C. The API 20 C AUX (bioMérieux, France) kit was used according to the manufacturer's instructions. Results were collected after 48 and 72 h, respectively. Results were analyzed manually following the manufacturer's instructions or using the apiweb software (bioMérieux, France).

### 2.4. DNA Extraction

For DNA extraction, fresh colonies were collected upon culturing the samples on PDA for 48 h at 28°C. Extraction was performed using the NucleoSpin Tissue (Macherey-Nagel, Germany) kit according to the manufacturer's instructions. Lyticase (Sigma, USA) and sorbitol buffer (1.2 M sorbitol, 10 mM calcium chloride, 0.1 M Tris/Cl pH of 7.5, and 35 mM *β*-mercaptoethanol) were added in the lysis step to weaken the chitin cell wall. The extracted DNA was then stored at −20°C until needed.

### 2.5. Typing of the ITS Gene

Amplification of the Internal Transcribed Spacer regions ITS 1 and ITS 4 was accomplished by adding 2 *μ*L of the sample DNA lysate, 0.4 *μ*L (20 pmol/*μ*L) of each 5′-TCCGTAGGTGAACCTGCGG-3′ (forward) and 5′-TCCTCCGCTTATTGATATGC-3′ (reverse), 9.7 *μ*L deionized water, and 12.5 *μ*L (250 U) of the AmpliTaq Gold PCR Master Mix (Applied Biosystems) [11]. The PCR thermal cycling conditions were 95°C for 12 min, 30 cycles of 95°C for 30 s, 54°C for 30 s, and 72°C for 100 s, and a final extension at 72°C for 10 min. 0.5 *μ*L of Exonuclease I (Thermo Scientific) and 1 *μ*L of Fast Alkaline Phosphatase (Thermo Scientific) were added to 6 *μ*L of presequencing PCR product in order to purify it. The thermal conditions for this step were 37°C for 15 min followed by 80°C for 15 min. ABI Prism BigDye Terminator v3.0 Ready Reaction Cycle Sequencing Kit (Applied Biosystems) was used to sequence the purified PCR product. The sequencing reaction was performed by adding 4 *μ*L of 5 X-diluted BigDye premix, 3 *μ*L of 1.2 *μ*M sequencing forward/reverse primers, and 3 *μ*L of the purified PCR product. PCR cycle was performed, consisting of initial denaturation step at 96°C for 1 min followed by 26 cycles of 96°C for 10 s, 50°C for 5 s, and 60°C for 4 min. BigDye X-Terminator Purification Kit (Applied Biosystems) was used to purify sequencing products according to the manufacturer's instructions. Sequencing plate was then loaded for sequencing electrophoresis on an ABI 3500 Avant Genetic Analyzer (Applied Biosystems). For sequence analysis, the CLC Main Workbench software v5.0 (CLC bio, Denmark) was used to assemble and align sequences and consensus sequences obtained were compared to ITS sequences in the GenBank database using BLASTn at the National Center for Biotechnology Information website ( http://blast.ncbi.nlm.nih.gov/Blast.cgi).

### 2.6. MLST

Thirty samples were chosen to be additionally typed using MLST. The 30 samples chosen where ITS identified as* C. albicans*. MLST was performed by amplification of 7 housekeeping genes (AAT1a, ACC1, ADP1, MPIB, SYA1, VPS13, and ZWF1B) as described by Shin et al. [14]. CLC Main Workbench software v5.0 (CLC bio, Denmark) was used to assemble and align sequences of the seven housekeeping genes and sequence types (STs) were determined by submitting the allelic profile of representative alleles to the MLST database (http://calbicans.mlst.net/).

### 2.7. Antifungal Susceptibility Testing

Antifungal susceptibility to 4 antifungal drugs, azoles (fluconazole and posaconazole), echinocandins (anidulafungin), and polyenes (amphotericin B), was performed. The minimum inhibitory concentrations (MICs) were determined using *E*-test strips (bioMérieux, France) following CLSI standards except for posaconazole and amphotericin B. No definite MIC was provided for posaconazole; accordingly MIC was determined as that of fluconazole since it belongs to the same category, while for amphotericin B the MIC used was 0.38 ug/mL [10]. RPMI 1640 with MOPS, glucose L-glutamine but no bicarbonate (AB Biodisk, bioMérieux, France) was the media of choice when performing the antifungal susceptibility testing. Media were prepared according to the manufacturer's instructions. After culturing of the samples in Potato Dextrose Broth (PDB), fungal suspension with 0.5 McFarland turbidity (or 10^5^ CFU/mL) was used to streak on the RPMI media. The strips were applied on the inoculated plate and incubated at 37°C for 48 h.* C. albicans* ATCC 90028 was used as a quality control.

### 2.8. Biofilm Formation Assay

Biofilm formation assay was performed on all 85 samples. Each sample was done in triplicate and the average was determined. Three to four colonies were suspended in YNB (Yeast Nitrogen Base, Fluka, Switzerland) and incubated overnight with gentle shaking. The optical density of each of the suspensions was adjusted to 0.65 [15]. 0.5 mL of the suspension was added to a flat-bottomed microtiter well (24-well plates, pretreated with 5% fetal bovine serum (BioWhittaker, Belgium)) at 4°C and placed in a shaker at 37°C for 3 h to allow for initial adhesion. Plates were then washed with 0.5 mL PBS buffer and another 0.5 mL of the cell suspension was added. Following 48 h incubation at 37°C, cells were washed with 1 mL PBS and fixed using 0.5 mL of 99% methanol for 15 min. Plates were then allowed to air-dry for 20 min. Staining was performed by adding 0.2% crystal violet, removed after 20 min, and followed by 0.75 mL of 33% acetic acid. The absorbance was immediately measured using a spectrometer (Thermo Spectronic) at 590 nm [15].* C. albicans* strain SC5314 was used as a reference strain.

### 2.9. Statistical Analysis

To determine statistical significance of the biofilm study, both a *t*-test and a post hoc ANOVA test were carried out. For the ANOVA test isolates were grouped into 3 groups: those with biofilm capabilities below the reference strain, those similar to the reference strain, and those above the reference strain. Statistical significance with the reference strain group was observed for both groups containing isolates above and below the reference strain (data not shown). A *P* value below 0.05 was deemed significant.

## 3. Results

### 3.1. Germ Tube versus API and CHROMagar

*C. albicans* is primarily identified in hospitals using germ tube formation. The germ tube test was repeated on all 85 samples, and our results matched the hospital identification. Based on germ tube testing, 62 samples (72.9%) were hospital-identified as* C. albicans*, 22 samples (25.9%) were identified as* Candida* non-*albicans*, and one sample (1.1%) was unidentifiable and needed further identification through typing.

For isolation and differentiation of major clinically significant* Candida* species, CHROMagar and API were used. Results showed that eight samples (9.4%) streaked on CHROMagar showed different identification than that shown using the germ tube test. On the other hand, 11 samples (12.9%) of those tested by API also did not match the germ tube results, while two samples (2.3%) did not show a match between germ tube testing when compared to the API and CHROMagar.

#### 3.1.1. ITS Sequencing

**Figure 1:**
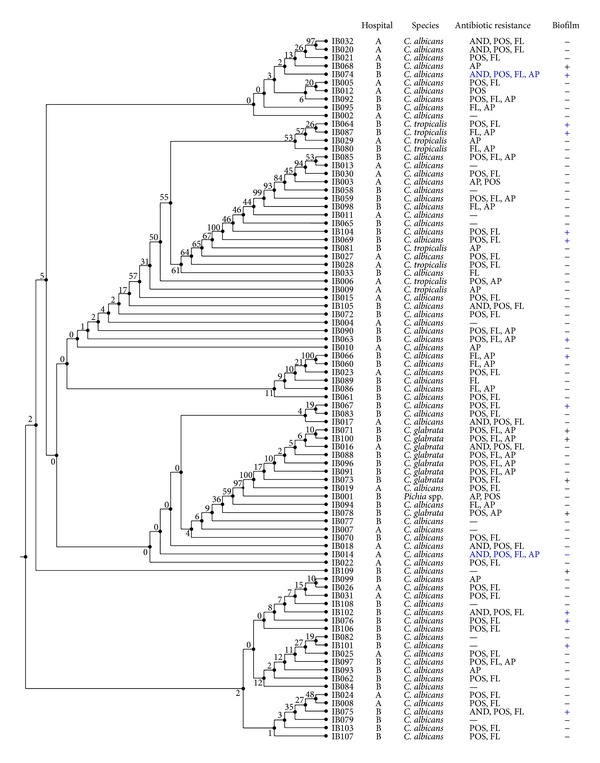
ITS neighbor-joining tree of all isolates collected. “+” denotes isolates that had biofilm forming capabilities above reference strain SC5314. AND, POS, FL, and AP refer to the antifungal drugs, anidulafungin, posaconazole, fluconazole, and amphotericin B, respectively.

Eighty-five samples were ITS sequenced. Results showed that 69 (81.1%) isolates were* C. albicans*, eight (9.4%) were* C. glabrata*, six (7%) were* C. tropicalis*, and one (1%) isolate belonged to* Pichia* spp. Among the* C. albicans* isolates, 18 samples (21.1%) were misidentified by the germ tube test, 13 samples (15.2%) were misidentified using cultivation on CHROMagar, and 13 (15.2%) were misidentified by API.  Table 1 summarizes the comparison between germ tube, API, CHROMagar, and ITS sequencing for those isolates showing discrepancies. The ITS sequences obtained in this study were aligned and a neighbor-joining tree was generated to monitor the clusters (Figure 1). The non-*albicans* species aligned together in two clusters apart from each other: one cluster containing most of the* C. glabrata* isolates and another that included most of the* C. tropicalis* ones. This indicated that ITS sequence in some* C. albicans* isolates has higher homology to non-*Candida* isolates than to those that belong to the same species. Leaw et al. showed that sequence analysis of the ITS region could not identify phylogenetically related species and uncommon yeast [11]. Typing of this region will tend to differentiate the isolates into major subclasses due to conserved sequences [10]. Also, isolates from hospitals A and B did not cluster together.

### 3.2. MLST

**Figure 2:**
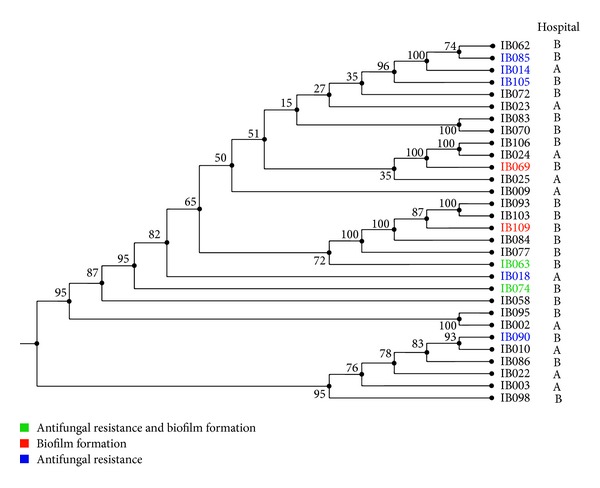
MLST tree. Tree represents 30 isolates chosen from each major subclass generated by ITS sequencing. Note the high bootstrap values implying a high level of confidence in the tree. MLST grouped the resistant strains next to each other implying high sequence relatedness such as that seen with IB085, IB014, and IB105. Furthermore strong biofilm producing strains such as IB109, IB077, IB063, and IB074 were also grouped close to one another. Multidrug resistance indicated here by color coding implies resistance to at least 3 out of the 4 drugs tested.

Out of the 85 ITS typed samples, 30 samples were chosen to be additionally typed using MLST. The 30 samples chosen were ITS-identified as* C. albicans*. The samples were chosen from each ITS cluster shown in Figure 1 and MLST was performed in order to compare between MLST and ITS neighbor-joining trees to determine whether ITS typing alone could be used to reflect strain relatedness. Moreover, the sequences of the seven housekeeping genes were concatenated into a single sequence and aligned against the other samples. The neighbor-joining tree was applied as shown in Figure 2. The 30 MLST typed samples did not have any ST representation on the website http://www.mlst.net. The sequences will be submitted to the creator of the online database, and new STs will be assigned. Four couples of isolates, IB083 and IB070, IB106 and IB024, IB093 and IB103, and IB095 and IB002, had a bootstrap value of 100 indicating their close relatedness. All other bootstrap values (with two exceptions) were above 50 implying a high level of confidence within the clades.

### 3.3. Antifungal Susceptibility Testing

**Figure 3:**
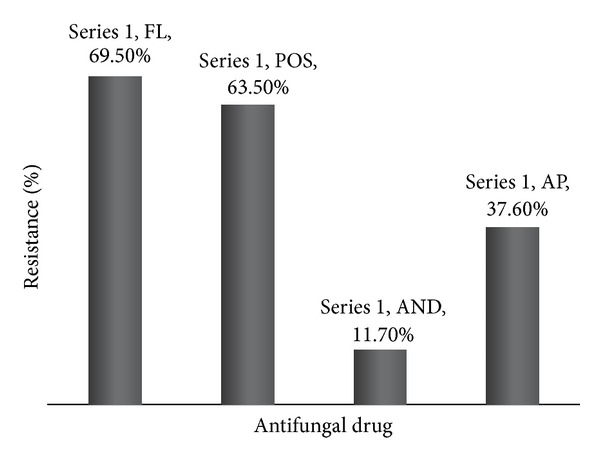
Drug resistance profile to four antifungal drugs. Note the high level of drug resistance observed.

Isolates were tested for antifungal susceptibility against the four antifungal drugs using the *E*-test. Fifty-nine isolates (69.5%) showed resistance against fluconazole followed by 54 being (63.5%) resistant against posaconazole and 32 (37.6%) resistant against amphotericin B. Although anidulafungin is a new antifungal agent, resistance was detected in ten of the isolates (11.7%) (Figure 3). Multidrug resistance was also considered, with 21 samples out of the 85 (25%) showing resistance against at least three antifungal agents (Figure 3). There was no statistically significant correlation between drug resistance and isolate location, or between resistance and hospital source. Most of the resistance isolates did come from urine samples, but since urine was the largest reservoir of isolates obtained, such a correlation is not significant.

### 3.4. Biofilm Formation

**Figure 4:**
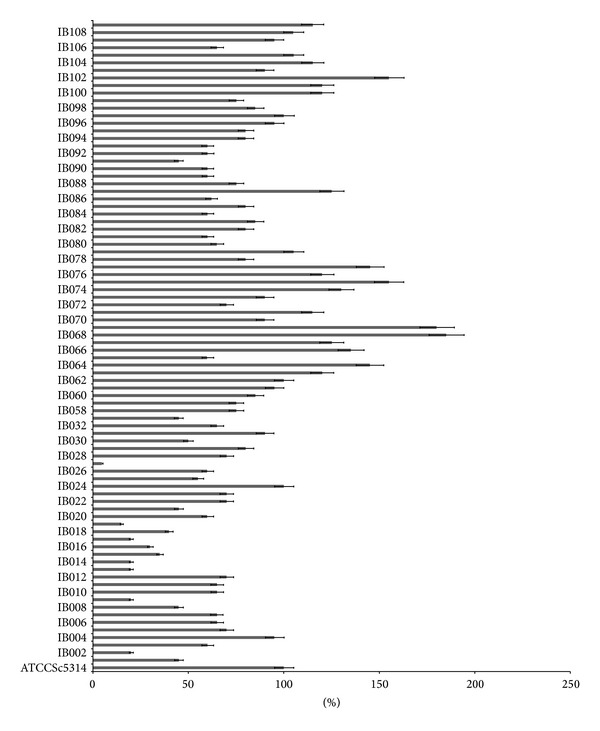
Biofilm formation. All 85 strains were assayed from biofilm formation and normalized to reference strain SC5314 (100%). 20% of isolates had higher biofilm forming capabilities than the reference strain. Bars represent standard deviation.

As can be seen in Figure 4, seventeen samples (20%) showed biofilm formation above levels of the wild-type reference strain SC5314 and were deemed to be strong biofilm formers. The *P* value was calculated using both Student's *t*-test and post hoc ANOVA test and was deemed significant if <0.05. No correlation between biofilm forming capabilities and hospitals was found. However correlation between biofilm, drug resistance, and phylogenetic relatedness was observed; isolates IB063 and IB074, which clustered together, were recovered from the same hospital and were strong biofilm formers and multidrug resistant.

## 4. Discussion

Identification of* Candida* at the species level is considered critical to provide proper treatment to severely ill patients [16]. In this study, 85 isolates of* Candida* species were collected from two major hospitals in Beirut/Lebanon between June and October 2011. Urine (36.4%) represented the main source of the collected isolates. Urinary tract is the main source of nosocomial infections and* Candida* species is the most common species among fungi recovered from urine [17], which was in harmony with our results.

Hospital identification of* Candida* species is based on the germ tube test that differentiates between* C. albicans* and* Candida* non-*albicans*. The germ tube is a rapid and cheap identification test of* C. albicans* that can be completed in 90–180 min. Germ tube formation is considered a pathogenicity factor in* C. albicans* [18]. Germ tube was performed on all 85 samples in an attempt to replicate the hospital results. This test is not considered an accurate test since it relies solely on phenotypic changes. Some* Candida* non-*albicans* species share similar features to that found in* C. albicans*, such as* C. dubliniensis* which also forms germ tube in serum. This fact contributes to misidentification incidences reported when germ tube is solely considered in hospitals [19]. Moreover,* C. africana* can also produce germ tube in serum and would be misidentified as* C. albicans* [20]. Such false-positive results might lead to the use of inappropriate antifungal agents especially that different* Candida* species have different innate primary resistance to antifungal drugs, resulting in hospitalization of the patient for a longer period of time. Yazbek et al. reported misidentification rate of 24% by the germ tube testing compared to real-time PCR for isolates collected from four major hospitals in Beirut, which was comparable to the 21.2% misidentification rates observed in this study [21].

Another conventional identification method that is being used is CHROMagar. This medium relies on the ability of different* Candida* species to form pigmented colonies due to the breakdown of substrates by enzymes of the fungus resulting in the change of color [16]. It is a selective and differential method for direct identification and isolation [22]. In this study, four out of all the tested isolates showed discrepancies in identification results between germ tube and CHROMagar; three identified as* C. albicans* by the germ tube were identified as* C. tropicalis* (two samples) and* C. glabrata* (one sample) by CHROMagar, and one identified as* C.* non-*albicans* using germ tube was identified as* C. albicans* on CHROMagar. These results showed that identification by these two methods can be contradictory and additionally emphasizes the need for alternative identification methods. A study in France revealed that* C. tropicalis* is considered the main, but not exclusive, source of false-positive identifications on chromogenic media [23], while Eraso et al. reported misidentification using CHROMagar and presence of both false-positive and false-negative results [16].

On the other hand, four of the isolates identified as* C. albicans *according to the hospital records were found to be* C. tropicalis, C. glabrata*, and* C. sphearical* based on API results, five identified as* C.* non-*albicans* were* C. albicans*, and one isolate not identifiable neither by CHROMagar nor by the hospital was identified as *C. sphaerical*. This method is considered reliable in the sense of sensitivity, but it is time consuming as some isolates are slow growers and will need 72 h for confirmation of the results [24]. Studies showed that the sensitivity of this method in identifying common yeast isolates was 96.3% with 66% identification for unconventional yeast isolates. Most studies confirmed the need for other identification methods along with the API based system to avoid errors and misidentifying isolates [24].

To assess the feasibility of using those conventional methods, we have sequenced the ITS gene of all isolates. ITS sequencing showed that conventional methods gave in many instances contradictory and inaccurate results. The sequence-based methods have been used for identification of* Candida* species and considered to be accurate and fast [9]. Moreover, sequence-based identification techniques were found to be more efficient than other phenotypic methods, and sequence analysis of the ITS region provided a higher percent of accurate identification [25]. Furthermore, a study conducted in UK showed that ITS sequencing was able to accurately identify isolates such as* C. africana* that could not be properly identified by conventional methods [20].

MLST was performed on 30 samples chosen from each major subclass generated by ITS sequencing. In contrast to ITS, MLST is based on sequence analysis of seven unrelated genes that are not conserved [26]. A neighbor-joining tree was generated for the samples typed and compared to that of the ITS. The relatedness of the samples differed in each tree. Tavanti et al. showed that MLST could be used for epidemiological studies due to its high discriminatory power among closely related species and isolates and the high reproducibility which other techniques lack [26]. Additionally,* C. albicans* isolates might undergo microevolution, which means that the same isolate can undergo a small variation in its genome to adapt to some situations such as resistance to antifungal drugs. These small changes in the genome would be very difficult to be recognized using typical sequencing techniques. MLST can identify these microevolutionary changes to elucidate the source of the transmission of the isolate and the evolutionary events in outbreaks or recurrent infections. MLST could be used for epidemiological studies whereas ITS sequencing is accurate for species identification only. The sequences of the 30 samples typed were submitted to the database (http://www.mlst.net) to assign the diploid sequence type (DST); however, they could not be identified by the database. The sequences will be sent to the database curators to assign new DSTs and add these sequences to the database.

Biofilm forming ability is an important factor that contributes to virulence of a pathogen. All 85 isolates were tested for biofilm formation, and 17 (20%) showed the ability to form biofilm at a higher rate than the reference strain SC 5314. These results were correlated to the ITS and MLST neighbor-joining trees to determine whether these isolates would cluster together. There was no observed correlation between ITS clusters and biofilm formation, while MLST clustered the six biofilm forming isolates (all from the same hospital) close to each other. Samples IB069 and IB070 are located in a subcluster with significant bootstrap value, while IB109, IB077, IB063, and IB074 are located in the same subcluster close to one another indicating that these samples share homology and sequence similarity. This correlation has never been shown before between a virulence trait and MLST sequence types. As such, this is an interesting finding whereby, in theory, virulence attributes for uncharacterized isolates could be predicted by MLST clustering.

Susceptibility to antifungal drugs was determined and the results revealed that 59 of the isolates (69.5%) were resistant to fluconazole, 54 (63.5%) to posaconazole, and 32 (37.6%) to amphotericin B. There was a significant difference from what was previously reported by Basma et al. in a study conducted in Lebanon on antifungal susceptibility of 116* C. albicans* hospital isolates [10]. The percentage of fluconazole (FL) resistance was 5.2% and 12.1% for IT (itraconazole). The resistance for the azole and especially FL has increased enormously perhaps due to uncontrolled use and overusage. On the other hand, the reported resistance by Basma et al. for amphotericin B (AP) was 1.7%, while in this study it was 38%, a 22-fold increase in five years [10]. These results indicated the rapid increase in the resistance for AP in clinical isolates even though usage is limited due to toxicity. The study done by Basma et al. also determined resistance against caspofungin (CS), which belongs to the most recent antifungal class of drugs, and results revealed that all of the tested isolates were susceptible [10]. However, in this study resistance to anidulafungin (AND), belonging to the same class of antifungals, was 11.9%. This showed again the emergence of resistance against this new class of antifungals. Additionally, 25% of the samples were resistant to three antifungals and above with two of the samples being multidrug resistant. The high misidentification rates can explain the high rate of drug resistance, since misidentification leads to inappropriate antifungal treatment. MLST grouped the resistant strains next to each other implying high sequence relatedness such as that seen with IB085, IB014, and IB105, located next to one another, and with IB063, IB018, and IB074. Only IB090 was found in another independent cluster. These findings show that resistant strains were phylogenetically related.

Interestingly, there was a correlation between antifungal susceptibility and biofilm formation since IB074 and IB063 (both collected from hospital B) had both of these features.* Candida* cells growing in a biofilm are known to be highly resistant to antifungal drugs through different mechanisms including active extrusion through efflux pumps [27]. These two samples, however, were related based on MLST typing indicating that they shared homology in more than one aspect, implying a possible case of microevolution of one strain and possible dissemination into another patient. Interestingly however IB063 as opposed to IB074 was not resistant to anidulafungin, which might be explained by microevolution resulting in loss/gain of drug resistance. However, since only two isolates exhibited such correlation and in light of the relatively low number of isolates that were sequenced by MLST, such a correlation cannot be further substantiated.

## 5. Conclusion

In conclusion this study has shown that the germ tube test method used by most hospitals is fairly inaccurate in identifying* Candida* species and as such either a combination of conventional methods or the use of molecular sequencing methods should be used. Such a high misidentification rate has resulted in unacceptably high rates of drug resistance, an issue that should be immediately addressed. Furthermore, this study is the first of its kind that attempts to correlate between strain relatedness, drug resistance, and a virulence attribute such as biofilm formation and found that most resistant strains grouped together. The drawbacks of this study are the relatively small number of samples and the lack of patient clinical history, which precluded any discussion of nosocomial infection.

## Figures and Tables

**Table 1:** Discrepancy in identification rates. Table encompasses the 22 isolates that were differentially identified by the four identification methods used.

Sample ID	Germ tube (hospital identification)	Germ tube	CHROMagar	API	ITS	Antifungal resistance*	Biofilm
IB001	Typing needed	−	Unidentifiable	*C. sphaerical *	*Pichia *spp.	AP, POS	−
IB016	*C. albicans *	+	*C. albicans *	*C. albicans *	*C. glabrata *	AND, POS, FL	−
IB019	Non-*albicans *	−	*C. glabrata *	*C. glabrata *	*C. albicans *	POS, FL	−
IB026	Non-*albicans *	−	*C. glabrata *	*C. glabrata *	*C. albicans *	POS, FL	−
IB028	*C. albicans *	+	*C. albicans *	*C. albicans *	*C. tropicalis *	POS, FL	−
IB029	*C. albicans *	+	*C. tropicalis *	*C. tropicalis *	*C. tropicalis *	AP	−
IB030	*C. albicans *	+	*C. tropicalis *	*C. tropicalis *	*C. albicans *	POS, FL	−
IB060	Non-*albicans *	−	*C. albicans *	*C. albicans *	*C. albicans *	FL, AP	−
IB063	Non-*albicans *	−	*C. tropicalis *	*C. tropicalis *	*C. albicans *	POS, FL, AP	+
IB064	*C. albicans *	+	*C. albicans *	*C. albicans *	*C. tropicalis *	POS, FL	+
IB067	Non*-albicans *	−	*C. albicans *	*C. albicans *	*C. albicans *	POS, FL	+
IB068	Non*-albicans *	−	*C. albicans *	*C. albicans *	*C. albicans *	AP	+
IB070	Non*-albicans *	−	*C. glabrata *	*C. glabrata *	*C. albicans *	POS, FL	−
IB071	Non-*albicans *	−	*C. tropicalis *	*C. tropicalis *	*C. glabrata *	POS, FL, AP	+
IB072	Non-*albicans *	−	*C. albicans *	*C. albicans *	*C. albicans *	POS, FL	−
IB077	Non-*albicans *	−	*C. albicans *	*C. albicans *	*C. albicans *	Sensitive to all	−
IB078	*C. albicans *	+	*C. glabrata *	*C. glabrata *	*C. glabrata *	POS, AP	+
IB080	Non-*albicans *	−	White color	*C. lusitaniae *	*C. tropicalis *	FL, AP	−
IB081	Non-*albicans *	−	*C. glabrata *	*C. glabrata *	*C. tropicalis *	AP	−
IB083	Non-*albicans *	−	*C. albicans *	*C. albicans *	*C. albicans *	POS, FL	−
IB086	Non-*albicans *	−	*C. albicans *	*C. albicans *	*C. albicans *	FL, AP	−
IB097	Non-*albicans *	−	*C. glabrata *	*C. glabrata *	*C. albicans *	POS, FL, AP	−

*AND: anidulafungin, AP: amphotericin B, FL: fluconazole, and POS: posaconazole. Biofilm “−” refers to strains that form biofilm at a rate lower than the reference strain SC5314, while biofilm “+” refers to strain that forms biofilm at a higher rate than the reference strain.
